# Environmental Stimulus on Stem Cell Behaviour

**DOI:** 10.1155/2018/4910185

**Published:** 2018-02-18

**Authors:** Eftekhar Eftekharpour, Josef Buttigieg

**Affiliations:** ^1^Regenerative Medicine Program, Spinal Cord Research Centre, Department of Physiology and Pathophysiology, University of Manitoba, Winnipeg, MB, Canada R3E 0J9; ^2^Department of Biology, Faculty of Science, University of Regina, Regina, SK, Canada S4S0A2

Stem cells are unique in their ability of self-renewal as well as being able to differentiate into multiple cell types. Due to this unique physiology, stem cells are often studied in the context of regenerative medicine, as they can replace lost or damage cells, thus potentially reversing deficits encountered during disease or injury. Furthermore, stem cells play a unique ability during the development of different tissue systems. Pools of these cells serve to provide the progenitor cells required to differentiate into mature cells. Although there is much potential not only in regenerative medicine but also in terms of understanding, the development of various tissues, many key gaps exist in our understanding of stem cell physiology.

This special issue has the main goal of further advancing the field of stem cell biology by presenting research articles that examine not only stem cell biology but also the environment that they reside in. The environment surrounding stem cells is thought to play a key role in not only regulating their self-renewal process but also in influencing their differentiation fates. These environmental cues can arise from a variety of sources. Signalling molecules, both from autocrine and paracrine sources, can be derived from hormones, ATP, and even neurotransmitters.

In the living organism, stem cells reside in environments that experience significantly lower PO_2_ values compared to the external atmospheric content. This fact, in addition to the special metabolic requirements of undifferentiated cells, likely plays a critical role in stem cell biology. Changes in PO_2_ due to development or pathology can result in the activation of a variety of genes involved in both metabolism and development. Related to changes in PO_2_ values is the production of reactive oxygen species (ROS). O_2_ acts as the terminal electron acceptor in the mitochondrial electron transport chain. Due to changes in O_2_ environment or inhibition of the ETC, there can be an increase or decrease in ROS production. Due to the extra electron, ROS can induce a variety of changes in the cell by altering gene transcription or even altering protein function.

It is our hope that these articles presented here will help not only answer current questions on stem cell biology but also guide future research.

